# Association Between Acute Alcohol Use and Firearm-Involved Suicide in the United States

**DOI:** 10.1001/jamanetworkopen.2023.5248

**Published:** 2023-03-29

**Authors:** Shannon Lange, Huan Jiang, Mark S. Kaplan, Kawon Victoria Kim, Jürgen Rehm

**Affiliations:** 1 Institute for Mental Health Policy Research, Centre for Addiction and Mental Health, Toronto, Ontario, Canada; 2 Campbell Family Mental Health Research Institute, Centre for Addiction and Mental Health, Toronto, Ontario, Canada; 3 Department of Psychiatry, University of Toronto, Toronto, Ontario, Canada; 4 Dalla Lana School of Public Health, University of Toronto, Toronto, Ontario, Canada; 5 Luskin School of Public Affairs, University of California, Los Angeles, Los Angeles; 6 Faculty of Medicine, Institute of Medical Science, University of Toronto, Toronto, Ontario, Canada; 7 Program on Substance Abuse, Public Health Agency of Catalonia, Barcelona, Spain; 8 Institute of Clinical Psychology and Psychotherapy, Technische Universität Dresden, Dresden, Germany; 9 Center for Interdisciplinary Addiction Research, Department of Psychiatry and Psychotherapy, University Medical Center Hamburg-Eppendorf, Hamburg, Germany; 10 I.M. Sechenov First Moscow State Medical University (Sechenov University), Moscow, Russian Federation

## Abstract

**Importance:**

Firearms are the method of suicide used most often in the US. Acute alcohol use is associated with an increased risk of suicide by firearm. However, the dose-response association between acute alcohol use and the probability of using a firearm as the method of suicide is unknown.

**Objective:**

To evaluate the association between the amount of alcohol consumed and the probability of using a firearm as the method of suicide.

**Design, Setting, and Participants:**

This cross-sectional study used mortality data from the US National Violent Death Reporting System on suicide decedents aged 18 years or older with a positive blood alcohol concentration (BAC; ie, ≥0.01 g/dL). Statistical analysis was performed from January 2003 to December 2020.

**Exposure:**

Acute alcohol use, ascertained via postmortem toxicologic examination.

**Main Outcomes and Measures:**

Probability of using a firearm as the method of suicide compared with all other methods of suicide.

**Results:**

The study included 45 959 male suicide decedents (mean [SD] age, 42.6 [14.8] years) and 12 136 female suicide decedents (mean [SD] age, 44.2 [13.8] years) with a positive BAC; of those, 24 720 male decedents (53.8%) and 3599 female decedents (29.7%) used a firearm as the method of suicide. The probability of using a firearm as the method of suicide when alcohol is consumed was higher for male decedents, with the probability starting at just below 0.50 and increasing to approximately 0.75. In contrast, for female decedents, the probability began at just above 0.30 and increased to approximately 0.55. For both male and female decedents, the dose-response curves were an inverted U shape; as BAC increased, the probability of firearm-involved suicide initially increased and then decreased at very high BACs (approximately 0.40 g/dL for male decedents and approximately 0.30 g/dL for female decedents; these BACs were present among only a small percentage of alcohol-involved suicides: male decedents, 589 [1.3%]; female decedents, 754 [6.2%]).

**Conclusions and Relevance:**

This cross-sectional study of suicide decedents who had consumed alcohol prior to their death suggests that, as alcohol consumption increased, the probability of a firearm-involved suicide increased until a certain BAC, at which point the probability started to decrease.

## Key Points

**Question:**

Does the probability of using a firearm as the method of suicide increase as the amount of alcohol consumed increases?

**Findings:**

In this cross-sectional study of 58 095 suicide decedents, the probability of a firearm-involved suicide increased as blood alcohol concentration increased until the blood alcohol level reached approximately 0.40 g/dL for male decedents and approximately 0.30 g/dL for female decedents, at which point the probability started to decrease.

**Meaning:**

This study suggests that interventions targeting heavy alcohol use may aid in efforts to reduce the suicide mortality rate, particularly suicides involving a firearm.

## Teaser

This cross-sectional study uses mortality data from the National Violent Death Reporting System to evaluate the association between the amount of alcohol consumed and the probability of using of a firearm as the method of suicide.

## Introduction

In 2021, more than 47 000 people died by suicide in the United States,^1^ more than 50% of whom used a firearm.^2^ The firearm-involved suicide rate in 2021 (among individuals ≥10 years of age, 8.75 per 100 000 population) was the highest since 1990.^2^ Research indicates that suicides by firearm are more likely to have been preceded by alcohol use than suicides by other methods^3,4^ and that the alcohol-firearm-suicide connection may be the strongest for individuals who acutely consume high amounts of alcohol.^5^ In 2013, Kaplan and colleagues^3^ found that among the general US population, acute alcohol intoxication (defined as a blood alcohol concentration [BAC] ≥0.08 g/dL) was associated with increased odds of using a firearm as the method of suicide for both male and female decedents (odds ratio, 1.76 [95% CI, 1.61-1.93] for male decedents and 1.68 [95% CI, 1.46-1.93] for female decedents).

Although the alcohol-firearm-suicide connection is well established in the current literature, our understanding of this association remains relatively vague. Specifically, it remains unknown whether there is a dose-response association between alcohol use and firearm-involved suicide. Therefore, the present investigation aimed to evaluate the association between the amount of alcohol consumed and the probability of using a firearm as the method of suicide. Based on the categorical evidence in the current literature, we hypothesized that there would be a monotonic increase in the probability of using a firearm as the method of suicide as the amount of alcohol consumed increased.

## Methods

This cross-sectional study used restricted-access mortality data from the US National Violent Death Reporting System (NVDRS).^6^ The present analyses were restricted to solitary suicides (ie, those in which there was a single person) among persons 18 years of age or older. The amount of alcohol consumed was derived from the BAC of decedents for whom a postmortem toxicologic examination was performed. The NVDRS restricted-access database is a deidentified data set containing incident-level data on violent deaths. Approval for the present study was obtained from the Centre for Addiction and Mental Health Research Ethics Board. This study was prepared with adherence to the Strengthening the Reporting of Observational Studies in Epidemiology (STROBE) reporting guideline for cross-sectional studies.

Statistical analysis was performed from January 2003 to December 2020. Based on the recommendation of the NVDRS, cases with a BAC higher than 0.60 g/dL were excluded, as such a value is highly unlikely and thus suspected to be in error.^7^ The probability of using a firearm as the method of suicide was computed in association with all other methods of suicide. Sex-specific generalized logistic regression models were applied to all suicide decedents with a positive BAC (ie, ≥0.01 g/dL). Linear, quadratic, and nonlinear associations were modeled. Results from the model with the lowest Akaike information criterion are reported. A nonlinear association was modeled using piecewise cubic polynomials. All models controlled for age, marital status, educational level, and race and ethnicity. Analyses were performed using R, version 4.0.5 (R Group for Statistical Computing).

## Results

The present analyses included 45 959 male suicide decedents (mean [SD] age, 42.6 [14.8] years) and 12 136 female suicide decedents (mean [SD] age, 44.2 [13.8] years) with a positive BAC (Table); of those, 24 720 male decedents (53.8%) and 3599 female decedents (29.7%) used a firearm as the method of suicide. The mean (SD) BAC was 0.14 (0.10) g/dL for male decedents and 0.15 (0.10) g/dL for female decedents.

**Table.** Characteristics of Suicide Decedents 18 Years of Age or Older Who Consumed Alcohol Prior to Their Death, NVDRS, 2003-2020

Characteristic	Decedents, No. (%)^a^	
	Female (n = 12 136 [20.9])	Male (n = 45 959 [79.1])
Age, mean (SD), y	44.2 (13.8)	42.6 (14.8)
Educational level		
Secondary school or GED certification or less	4457 (36.7)	21 399 (46.6)
Some postsecondary school	1950 (16.1)	6416 (14.0)
Associate’s or bachelor’s degree	2553 (21.0)	6656 (14.5)
Graduate degree	687 (5.7)	1597 (3.5)
Not available	2489 (20.5)	9891 (21.5)
Race and ethnicity		
African American or Black, non-Hispanic	524 (4.3)	2566 (5.6)
Hispanic	776 (6.4)	3601 (7.8)
White, non-Hispanic	10 153 (83.7)	37 652 (81.9)
Other, non-Hispanic^b^	683 (5.6)	2140 (4.7)
Marital status		
Married, civil union, or domestic partnership	3792 (31.2)	14 212 (30.9)
Separated, widowed, or divorced	4650 (38.3)	13 002 (28.3)
Single or never married	3694 (30.4)	18 745 (40.8)
Blood alcohol concentration, tertiles, g/dL		
0.011-0.199	8839 (72.8)	32 471 (70.7)
0.20-0.399	3130 (25.8)	12 899 (28.1)
0.40-0.60	167 (1.4)	589 (1.3)

Abbreviations: GED, General Educational Development; NVDRS, National Violent Death Reporting System.

^a^ Percentages may not add to 100% due to rounding.

^b^ Refers to all races other than African American or Black and White and persons who are non-Hispanic (not further defined by the NVDRS).

Overall, the probability of using a firearm as the method of suicide when alcohol was consumed was higher for male decedents, with the probability starting at just below 0.50 and increasing to approximately 0.75. In contrast, for female decedents, the probability began at just above 0.30 and increased to just below 0.55 (Figure). A nonlinear model best described the association between BAC and the probability of firearm-involved suicide. Specifically, for both male and female decedents, the dose-response curves were an inverted U shape; as BAC increased, the probability of firearm-involved suicide initially increased, then decreased after a BAC of approximately 0.40 g/dL for male decedents and a BAC of approximately 0.30 g/dL for female decedents. These BACs were associated with only a small percentage of alcohol-involved suicides (male decedents, 589 [1.3%]; female decedents, 754 [6.2%]) (Table).

**Figure.**
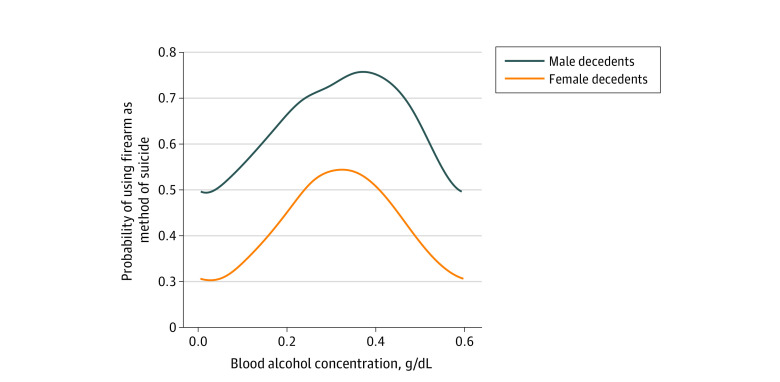
Dose-Response Curve for Blood Alcohol Concentration (BAC) and Probability of Using a Firearm as the Method of Suicide Among Male and Female Decedents Among female suicide decedents (n = 12 136), 8839 (72.8%) had a BAC of 0.011 to 0.199 g/dL, 3130 (25.8%) had a BAC of 0.20 to 0.399 g/dL, and 167 (1.4%) had a BAC of 0.40 to 0.60 g/dL. Among male suicide decedents (n = 45 959), 32 471 (70.7%) had a BAC of 0.011 to 0.199 g/dL, 12 899 (28.1%) had a BAC of 0.20 to 0.399 g/dL, and 589 (1.3%) had a BAC of 0.40 to 0.60 g/dL.

## Discussion

The present findings suggest that, as the amount of alcohol consumed increases, the probability of using a firearm as the method of suicide increases until a certain BAC level (approximately 0.40 g/dL for male decedents and 0.30 g/dL for female decedents), at which point the probability starts to decrease. Although the exact BAC depends on the time period during which alcohol is consumed, female and male individuals with average body weights of 72.6 and 81.6 kg, respectively, would have to drink more than 10 drinks to reach such BAC levels (based on the Widmark formula^8^). Only at very high doses of alcohol does the probability of a firearm-involved suicide begin to decrease compared with other methods, and only a very small percentage of male and female suicide decedents had BACs this high. This decrease in probability could be because when such large amounts of alcohol are consumed, individuals have a lower degree of motor coordination^9^ and are less capable of operating a firearm; this postulation has also been put forth by others.^10^ This supposition would also explain why the dose-response curve for female decedents begins to decrease at a lower BAC compared with male decedents because female individuals achieve higher BACs and become more impaired than male individuals after drinking equivalent amounts of alcohol.^11^ However, the decreasing probability could also be an artifact of the relatively low number of decedents with a BAC at such high levels (eg, ≥0.40 g/dL, as shown in the Table).

A prevention strategy commonly brought up when discussing firearm-involved suicide is means restriction (ie, decreasing access to suicide means).^12^ Despite means restriction being an evidence-based approach to reducing suicide risk, the implementation of firearms restriction at the clinical level is not without its difficulties, as recently discussed by Betz and colleagues.^13^ However, the present findings suggest there could be an alternative avenue for preventing suicide that may be worth exploring—addressing hazardous and heavy alcohol use among individuals at risk of suicide. For instance, among most people who consume alcohol, if hazardous and heavy alcohol use is reduced, the probability of using a firearm as the method of suicide would also be reduced. Assuming the reduced consumption does not result in a prevented suicide altogether, it may result in an increased probability of using a less lethal method, for which there is a lower likelihood that the method would result in death. Thus, such incidents would constitute a suicide attempt, after which individual-level suicide prevention efforts could be used to prevent a subsequent attempt or potential death by suicide thereafter. Such strategies could include routine screening, brief interventions, and referral to treatment, when necessary. Most individuals (approximately 80%) who die by suicide engage with the health care system in the year prior to their death.^14^ Thus, such intervention strategies, coupled with suicide risk assessments, have a high coverage potential. However, the strategy of targeting alcohol use does not appear to be relevant for the heaviest drinkers.

### Strengths and Limitations

This study has some strengths. To our knowledge, this is the first study to evaluate the dose-response association between alcohol use and firearm-involved suicides. Another strength is the database used; the NVDRS provides BACs among suicide decedents for whom the specific method of suicide is also available. However, this study also has some limitations. A limitation of the NVDRS with respect to the present study is that toxicologic testing in the US depends greatly on local resources. As such, in places where resources are limited, toxicologic data are often collected only from decedents for whom this information is important for determining the cause of death. It is possible that when a firearm is involved, the cause of death is more easily identifiable than when another method is used. As such, the probability of firearm-involved suicide in the present analyses may be affected by this practice.

## Conclusions

In this cross-sectional study of suicide decedents who had consumed alcohol prior to their death, we found that the more alcohol consumed, the higher the probability of using one of the most lethal methods of suicide—a firearm (90% of suicide attempts involving a firearm will result in death^15^). This association suggests that scalable interventions targeting heavy alcohol use (eg, alcohol control policies) may potentially reduce the suicide mortality rate in the US, which appears to be increasing again after decreasing for the past 3 years.^1^

## Notes

Supplement.
Data Sharing Statement
