# Supplementary material for: Association Between Acute Alcohol Use and Firearm-Involved Suicide in the United States
Source: JAMA Netw Open. 2023 Mar 29;6(3):e235248. doi: 10.1001/jamanetworkopen.2023.5248 (PMC10061235; doi:10.1001/jamanetworkopen.2023.5248)
Supplement: Supplement. — Data Sharing Statement [file jamanetwopen-e235248-s001.pdf]

Data Sharing Statement

Lange. Association Between Acute Alcohol Use and Firearm-Involved Suicide in the United States. *JAMA Netw Open*. Published March 29, 2023.
doi:10.1001/jamanetworkopen.2023.5248

Data
Data available: No

Additional Information
Explanation for why data not available: The data that support the findings of this study are available from the Centers for Disease Control and Prevention, National Center for Injury Prevention and Control. Restrictions apply to the availability of these data, which were used under agreement for this study and thus, these data cannot be shared.
